# Co-Doped Porous Carbon/Carbon Nanotube Heterostructures Derived from ZIF-L@ZIF-67 for Efficient Microwave Absorption

**DOI:** 10.3390/molecules29112426

**Published:** 2024-05-21

**Authors:** Liming He, Hongda Xu, Yang Cui, Jian Qi, Xiaolong Wang, Quan Jin

**Affiliations:** 1 The Key Laboratory of Automobile Materials (Ministry of Education), School of Materials Science and Engineering, Jilin University, 5988 Renmin Street, Changchun 130022, China; helm22@mails.jlu.edu.cn (L.H.); cuiyang23@mails.jlu.edu.cn (Y.C.); 2 State Key Laboratory of Integrated Optoelectronics, College of Electronic Science and Engineering, International Center of Future Science, Jilin University, 2699 Qianjin Street, Changchun 130012, China; xuhd22@mails.jlu.edu.cn (H.X.); brucewang@jlu.edu.cn (X.W.); 3 State Key Laboratory of Biochemical Engineering, Institute of Process Engineering, Chinese Academy of Sciences, Beijing 100190, China

**Keywords:** two-dimensional MOFs, carbon nanotubes, microwave absorbing, micropores, heterostructures

## Abstract

Carbon-based magnetic metal composites derived from metal–organic frameworks (MOFs) are promising materials for the preparation of broadband microwave absorbers. In this work, the leaf-like co-doped porous carbon/carbon nanotube heterostructure was obtained using ZIF-L@ZIF-67 as precursor. The number of carbon nanotubes can be controlled by varying the amount of ZIF-67, thus regulating the dielectric constant of the sample. An optimum reflection loss of −42.2 dB is attained when ZIF-67 is added at 2 mmol. An effective absorption bandwidth (EAB) of 4.8 GHz is achieved with a thickness of 2.2 mm and a filler weight of 12%. The excellent microwave absorption (MA) ability is generated from the mesopore structure, uniform heterogeneous interfaces, and high conduction loss. The work offers useful guidelines to devise and prepare such nanostructured materials for MA materials.

## 1. Introduction

Microwave technology has been extensively utilised in civil, military, medical, and scientific research fields due to the rapid evolution of electronic devices as well as wireless communication instruments, which are directly related to national security and social development [1,2,3]. However, electromagnetic wave radiation poses potential threats to biological systems, information security, and electromagnetic compatibility. Designing efficient MA materials has become the most effective method to address this issue [4,5]. The ideal MA materials should meet the electromagnetic wave attenuation, impedance matching characteristics, and the practical application requirements such as light weight and thin coating [6,7,8]. In recent years, carbon materials like graphene [9,10] and carbon nanotubes [11,12] have gained much interest in many fields due to their low density, low price, and high conductivity. Nevertheless, the impedance mismatch of individual materials limits their applications, necessitating an urgent need for a method to obtain carbon-based composites with outstanding MA performance [13].

Zeolite imidazolate framework (ZIF-L) is a two-dimensional (2D) MOF that inherits the large specific surface area and tunable pore structure of MOFs, together with ultra-high carrier concentration and abundant active sites, unlike conventional MOF-derived carbon materials (e.g., MIL-101 [14], FeM-MIL-88B [15] MIL-125(Ti) [16], Co-MOF-74 [17], etc.), which have the disadvantages of high loading and large matching thickness. Porous carbon materials made from ZIF-L have recently been widely used in MA and have shown excellent performance. Examples include HCF@NC/Co [18], H-800 [19], CoZn/C@MoS_2_@PPy [20], etc., where HCF@NC/Co achieves an EAB of 6.56 only at a loading of 10%. However, ZIF-L tends to undergo unavoidable shrinkage, aggregation, and collapse during high-temperature pyrolysis, leading to structural damage and a reduction in the functionality, thus limiting its application. KCl [21], NiCl [6], and Ag(NO_3_)_2_ [22] have been previously used to modify ZIF-L in order to improve its MA performance. However, these processes are complex and alter the originally leaf-like shape form of ZIF-L. Therefore, it is necessary to explore a simpler and more convenient method that can retain the leaf-like foliated shape of ZIF-L following pyrolysis, while exhibiting good MA performance.

The MOF-on-MOF structure, including the core–shell MOF@MOF heterojunction, not only integrates the benefits of MOFs but also avoids structural collapse [23,24]. Compared with single MOFs, carbon/metal composites derived from dual MOF-on-MOF exhibit greater synergistic effect and dielectric loss performance [25]. For example, the ZnO/NPC@Co/NPC obtained by coating ZIF-67 onto the ZIF-8 surface followed by carbonization has a relatively excellent MA performance with an EAB of 4.2 GHz at a small matching thickness [26]. Furthermore, due to the catalytic action of Co atoms, the composites containing ZIF-67 generate carbon nanotubes on the surface during pyrolysis [27,28]. The carbon nanotubes and nanosheets have abundant interconnected pores and heterojunction interfaces. The use of a multi-scaled heterostructure can enhance multiple reflections, optimize the impedance matching, and present an efficient method of improving the MA performance of the material [29,30].

In this study, we propose a 2D carbon–carbon heterostructure based on the MOF-on-MOF nanostructure. The novelty of the article can be summarized in the following two points: firstly, ZIF-67 is uniformly coated on ZIF-L, and two-dimensional lobed Co-doped porous carbon-carbon nanotube heterostructures (DPC/CNTs) are obtained by thermal cleavage; secondly, the even distribution of ZIF-67 preserves the original structure of ZIF-L, ensures the uniform distribution of Co particles on ZIF-L, and promotes the interfacial polarization. When the loading amount of ZIF-67 is 2 mmol, DPC/CNTs exhibit an EAB of DPC/CNTs-2, which can reach 4.8 GHz at a thickness of 2.2 mm. In the thickness area from 1 to 4 mm, the effective absorption frequency reaches 6.32~18.0 GHz with an RL_min_ value of −42.2 dB. Notably, the absorption dose added to the paraffin is controlled to be around 12%, which is lower than most reported ZIF-based composite materials. The research findings offer new insights on the potential usage of 2D MOFs in the field of MA.

## 2. Experimental Section

### 2.1. Materials

Zinc nitrate hexahydrate (Zn(NO_3_)_2_·6H_2_O, 99%), cobalt nitrate hexahydrate (Co(NO_3_)_2_·6H_2_O, 99%), 2-methylimidazole (2-MeIm, 98%), methanol (≥99.5%), and ethanol (≥99.7%) were obtained from Aladdin Biochemical Technology established in 2008 in Shanghai, China. All chemicals were used directly without further treatment.

### 2.2. Experiments

**Scheme 1:**
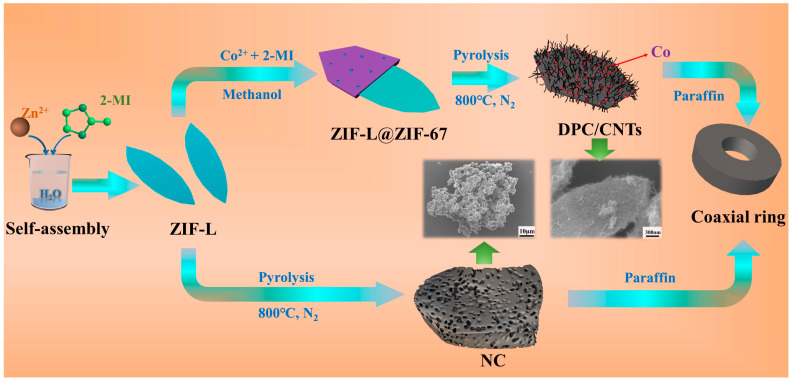
The synthesis process of the DPC/CNTs composites.

The procedure for the preparation of the DPC/CNTs is illustrated in Scheme 1, and the experimental method is described in the following.

#### 2.2.1. Synthesis of ZIF-L Precursors

For a general experiment, 3.8 g of 2-MeIm and 2.3 g of Zn(NO_3_)_2_·6H_2_O were dissolved separately in 100 mL of distilled water and named Solution A and B, respectively. Solution B was then slowly combined with solution A and stirred for 3 h to obtain a milky white precipitate. The mixture was subsequently centrifuged at 6000 rpm for 3 min, followed by washing with alternating deionised water and ethanol several times; then, it was dried in an oven at 60 °C for 12 h.

#### 2.2.2. Synthesis of ZIF-L@ZIF-67

ZIF-L@ZIF-67 was prepared in accordance with the literature [31]. A homogeneous solution was obtained by sonication and dispersion of 0.3 g of the ZIF-L precursor in 20 mL of methanol for 30 min. A solution was produced by adding 1 mmol of Co(NO_3_)_2_∙6H_2_O to 12 mL of methanol. In a separate solution, 6 mmol of 2-MeIm was dissolved entirely in methanol (20 mL). The two methanol solutions were then mixed, followed by the rapid addition of the ZIF-L precursor described above. The final mixture was then stirred for 10 h at about 25 °C. The violet precipitate was centrifuged, washed several times with methanol, and allowed to dry at 60 °C for 12 h. Co^2+^ (1, 2, 4, and 6 mmol) and 2-MeIm (6, 12, 24, and 36 mmol) were added, respectively. The thickness of the ZIF-67 shell could be adjusted by varying the additions.

#### 2.2.3. Carbonization of the Materials

Carbonization of ZIF-L@ZIF-67 was carried out in a tube furnace filled with nitrogen at a rate of 2 °C/min and a holding temperature of 800 °C, at which temperature the samples were kept for 3 h to allow complete carbonization. The as-prepared materials were named DPC/CNTs-1, DPC/CNTs-2, DPC/CNTs-4, and DPC/CNTs-6 according to the amount of loaded ZIF-67. The ZIF-L and ZIF-67 were carbonized in the same way, and the resulting samples were named NC and Co/C, respectively.

### 2.3. Characterization

Powder X-ray diffraction (XRD) was performed with an XRD-D/Max 2500 PC Rigaku with Cu Kα radiation (λ = 0.154 nm, scan step = 4°/min). Sample morphology and elemental composition were examined by field emission scanning electron microscopy (SEM) on a JSM-6700F (JEOL Ltd., Akishima, Tokyo, Japan) and transmission electron microscopy (TEM) on a JEM-2100F (JEOL Ltd., Akishima, Tokyo, Japan). For the TEM and EDS, the probe type was X-Max, the number of channels was 1024, the energy range (keV) = 40 keV, and the energy per channel (eV) = 40.0 eV. All of the above instruments were manufactured in Japan. Raman spectroscopy was performed by Virsa (Renishaw plc, Gloucestershire, UK, wavelength = 532 nm, spectral resolution < 2.5 cm^−1^, spatial resolution XY < 0.5 μm, Z < 2 μm). Nitrogen adsorption and desorption isotherms, as well as the pore size distribution, were determined using ASAP2460 (Micromeritcs, Norcross, GA, USA).

### 2.4. Electromagnetic Parameter Measurement

The relative complex permittivity and permeability of the materials over the 2–18 GHz band were measured using a vector network analyzer (VNA, MS46122B). The test specimens were prepared by mixing the prepared material homogeneously with paraffin wax, where the mass fraction of the material was 12 wt%. The mixture was pressed into coaxial rings (3.04 mm inner diameter and 7 mm outer diameter) with a thickness of 2 to 3 mm. The final MA performance was evaluated based on the transmission line theory and the metal backsheet model from the measured electromagnetic parameters using the formula below.

(1) $$RL=20\mathrm{lg}\left|\frac{Z_{\mathrm{in}\;}-Z_{0}}{Z_{\mathrm{in}\;}+Z_{0}}\right|$$

(2) $$Z_{in}=Z_{0}\sqrt{\frac{\mu_{r}}{\varepsilon_{r}}}\tanh\left(j\frac{2\pi fd}{c}\sqrt{\mu_{r}\varepsilon_{r}}\right)$$

where *Z*_in_ and *Z*_0_ represent the input impedance and free space impedance, respectively, while *ε*_r_, *μ*_r_, *f*, *d*, *c* represent the relative permittivity, permeability, frequency, thickness, and velocity of light, respectively.

## 3. Results and Discussion

**Figure 1:**
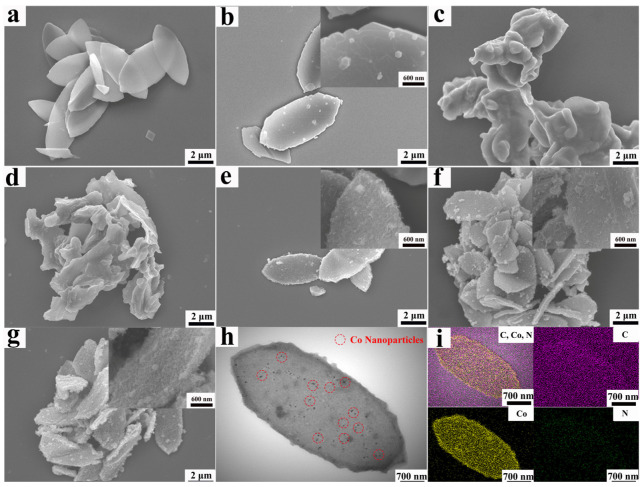
SEM images of (**a**) ZIF-L, (**b**) ZIF-L@ZIF-67-2, (**c**) NC, and (**d**–**g**) DPC-CNTs-1, 2, 4, 6; TEM images (**h**) and EDS mapping (**i**) of DPC-CNTs-2.

Firstly, the 2D leaf-like ZIF-L was synthesized through the coordinated interaction between Zn^2+^ and 2-MeIm. Subsequently, core-shelled ZIF-L@ZIF-67 nanosheets were constructed through the reaction between Co^2+^ and 2-MeIm. As shown in Figure 1a and Figure S1a, ZIF-L and ZIF-67 exhibited leaf-like and granular morphologies, consistent with previous work [32,33]. At the same time, a uniform ZIF-67 film can be clearly observed on the ZIF-L surface, causing the edges of the nanosheets to transition from the smooth surface of ZIF-L to an inclined surface (Figure 1b). It is noteworthy that with a low amount of loaded ZIF-67, no uniform covering layer is formed (as shown in Figure S1b). Conversely, an excessive reaction of Co^2+^ and 2-2-MeIm leads to the formation of a number of dodecahedral structures of ZIF-67 (Figure 1b and Figure S1c). The formation of Co nanoparticles occurred during the thermal decomposition process, accompanied by the generation of graphite carbon [34,35]. In addition, the pyrolysis products of ZIF-L@ZIF-67, ZIF-L and ZIF-67, were also characterized morphologically. After thermal decomposition, the colour of the samples shifted from purple or white to black. The ZIF-L@ZIF-67 maintained a good morphology, obtaining Co-doped porous carbon leaves (Figure 1e–g). However, ZIF-L@ZIF-67-1 experienced partial collapse owing to a lower loading amount, while the NC suffered collapse due to the evaporation of the Zn elements (Figure 1c), and the Co/C exhibited significant aggregation (Figure S1d). This indicates that the MOF-on-MOF synthesis strategy successfully prevented the potential collapse or aggregation of the ZIF-L 2D structure during thermal decomposition.

We observed the Co nanoparticles inside the material by TEM, as shown in Figure 1h, and the interface between Co and C was clearly seen under high-resolution transmission electron microscopy (HR-TEM) (Figure S2a). As shown in Figure S2a, three different types of crystal planes with d spacings of 0.21 nm, 0.30 nm, and 0.43 nm correspond to the (111), (200), and (220) crystal planes of metallic Co, respectively. In addition, energy dispersive X-ray spectroscopy (EDS) further demonstrated that DPC-CNT is mainly a composite material composed of elemental Co and elemental C (Figure 1i and Figure S2b), with cobalt uniformly distributed in the leaf-shaped material (Figure 1i). The uniform distribution of Co elements indicates that DPC-CNTs-2 inherits the advantage of uniform arrangement of MOFs atoms to a certain extent; at the same time, this also facilitates the generation of interfacial polarization. Additionally, under the catalytic action of cobalt elements in ZIF-67, distinct carbon nanotubes were observed on the surface of the carbon framework (Figure S1e) [28], which gradually became more densely packed with the increase in the ZIF-67 loading amount (Figure 1e–g). The carbon framework structure endows the samples with a highly active surface, while carbon nanotubes can generate more defects and enhance dipole polarization. Abundant interfaces between carbon nanotubes and porous carbon can enhance interface polarization, thereby improving the dielectric loss capacity and enhancing the MA performance of DPC/CNTs [36].

**Figure 2:**
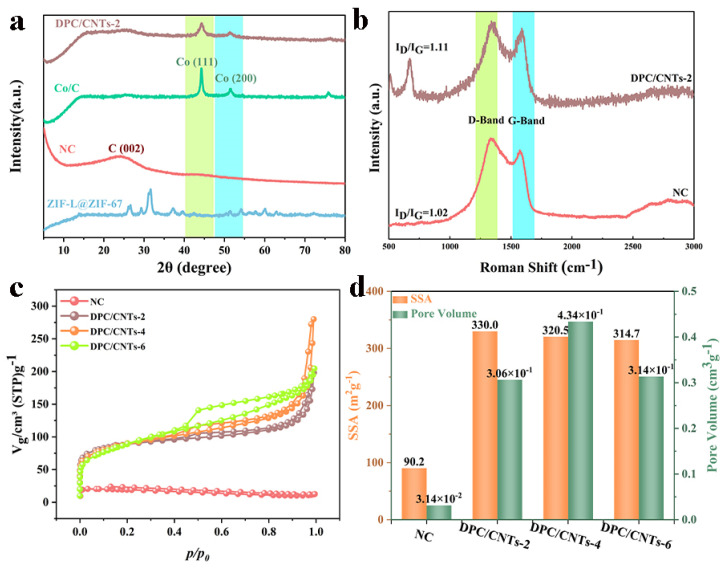
(**a**) XRD, (**b**) Raman spectra (**c**), N_2_ adsorption–desorption isotherms, and (**d**) corresponding SSAs and pore volumes of the ZIF-L@ZIF-67, NC, Co/C, and DPC/CNTs composites.

The crystal structures of ZIF-L@ZIF-67, NC, Co/C, and DPC/CNTs-2 were recognized by XRD (Figure 2a). In the XRD spectrum of ZIF-L@ZIF-67, the diffraction peaks corresponding to both ZIF-L and ZIF-67 phases were observed, indicating the successful synthesis of ZIF-L@ZIF-67. In the XRD curve of ZIF-L [32], a prominent peak was observed at about 25°, representing the (002) crystal plane of carbon [37]. The XRD spectra of Co/C and DPC/CNTs-2 showed broad diffraction peaks at around 44° and 51°, respectively, which correspond to the Co (111) and Co (200) crystal planes. This indicates the formation of metallic Co [38].

Raman spectroscopy can be employed to reflect the level of disorder in carbon materials [39]. The D peak (~1360 cm^−1^) represents the defects in the crystal structure of carbon atoms, while the G peak (~1580 cm^−1^) represents the in-plane stretching vibration of sp^2^ hybridized carbon atoms. The extent of the graphitisation of the sample is represented by the ratio of peak intensities (I_D_/I_G_). In Figure 2b, the I_D_/I_G_ ratios for NC and DPC/CNTs-2 are 1.11 and 1.02, respectively, indicating an improvement in the graphitisation with the addition of Co elements [40,41]. This is related to the strong catalytic ability of Co in promoting graphitisation. A stronger graphitisation intensity signifies that the DPC/CNTs-2 composite has a higher conductivity loss capacity compared to ZIF-L, which is crucial for enhancing the dielectric loss capacity of the materials.

To study the impact of the ZIF-67 content on the pore volume and specific surface area of the materials, nitrogen adsorption and desorption experiments were performed. As displayed in Figure 2c, NC the DPC/CNTs composites exhibit a typical Type II isotherm, and at smaller relative pressures (*P/P*_0_ < 0.1), the steeper slope of the curve suggests the existence of micropores [42], consistent with the pore size distribution (Figure S3). The specific surface area and pore volume of the four samples were statistically analysed, as shown in Figure 2d. The composites of the DPC/CNTs exhibited a greater specific surface area and larger pore volume in comparison to the NC. The rich porous structure and large specific surface area not only help to generate defect polarization but also effectively regulate the complex dielectric constant and optimize the impedance matching of MA materials, which is beneficial to allowing more electromagnetic waves to access the material and broaden the EAB of the material [43,44].

**Figure 3:**
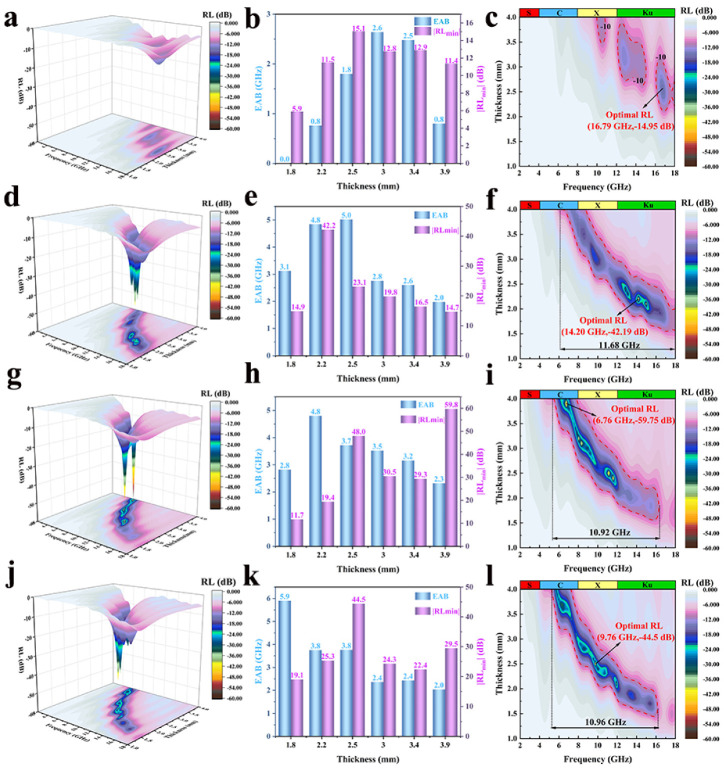
The RL and EAB values for NC and DPC/CNTs composites under a filling rate of 12 wt%, (**a**–**c**) NC, (**d**–**f**) DPC/CNTs-2, (**g**–**i**) DPC/CNTs-4, and (**j**–**l**) DPC/CNTs-6.

Figure 3 and Figure S3 show RL plots of the NC, Co/C, and DPC/CNTs with a filling rate of 12 wt%. For a clearer presentation of the results, the corresponding key parameters of RL_min_ and EAB for the materials are shown in Figure 3b,e,h,k. It is noteworthy that the RL_min_ is closely related to the loading amount of ZIF-67 and the thickness of the absorber. When only ZIF-67 or less ZIF-67 is present, the NC, Co/C, and DPC/CNTs-1 have poor MA properties due to the structural collapse caused by pyrolysis (Figure 3a–c and Figure S4). In contrast, DPC/CNT-2, 4, and 6 with higher ZIF-67 loading show better MA performance, and by adjusting the matched thickness between 1 and 4 mm, the EAB values of the materials can reach 11.68, 10.92, and 10.96 GHz, respectively. Among them, the RL_min_ of DPC/CNTs-4 at 6.76 GHz is −59.75 dB (Figure 3g–i). When the matched thickness of DPC/CNTs-2 is 2.2 mm, which corresponds to an EAB of 4.84 GHz, the RL_min_ is −42.19 (Figure 3d–f). The optimized MA performance of DPC/CNTs indicates that the well-maintained morphology and the generation of carbon nanotubes on the surface enhance the reflection loss and broaden the EAB as well. Even at a low filling rate, it is easy to establish a conductive network inside the polymer, achieving better electromagnetic absorption. Meanwhile, the increased surface and interface brought by the carbon/carbon heterostructure also enhance the effective absorption bandwidth.

**Figure 4:**
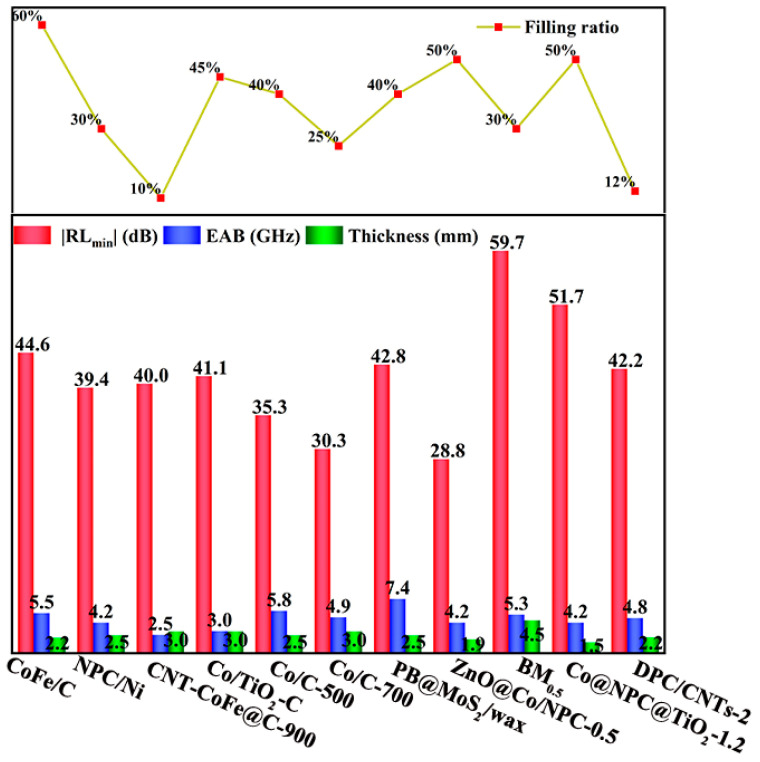
Comparison of the filling ratio, RL_min_, and EAB of reported MA materials over the frequency band from 2 to 18 GHz.

The MA performance of DPC/CNTs-2 was compared with other previously reported MOF-based nanomaterials in terms of the filling rate, thickness, minimum RL value, and EAB [26,45,46,47,48,49,50,51,52] (Figure 4). The results indicate that almost all Co-based MOF materials exhibit excellent MA performance, among them, BM 0.5 and Co@NPC@TiO_2_ achieve RL_min_ values of −59.7 and −51.7, respectively, due to their outstanding structural and compositional design. PB@MoS_2_/wax, benefiting from the synergistic interaction between different components, achieves an absorption bandwidth of 7.4 GHz. However, most of the materials have loading amounts above 30 wt%, and the DPC/CNTs-2 in our work demonstrates a high absorption peak and a wide effective absorption bandwidth at 12 wt% loading amount, which highlights its enormous potential as a high-performance MA material.

**Figure 5:**
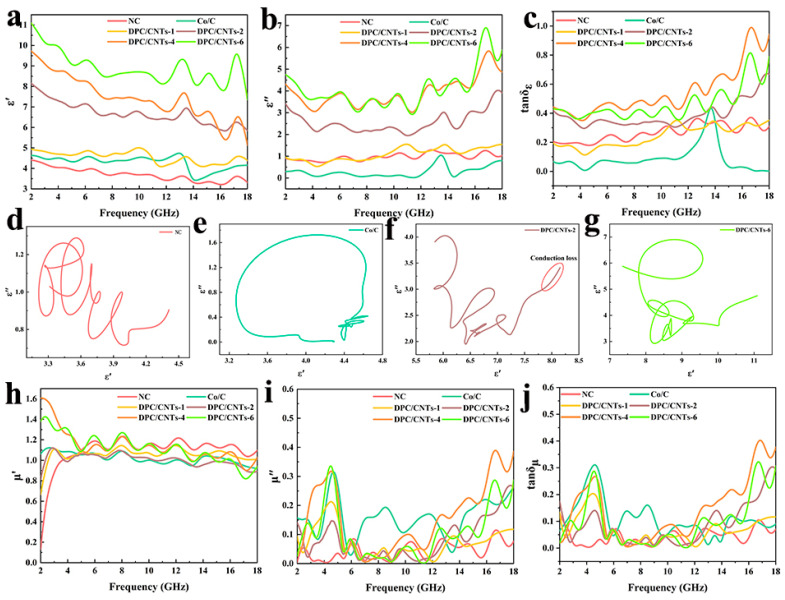
EM parameters of the NC, Co/C, and DPC/CNTs composites: (**a**,**b**) complex permittivity, (**c**) dielectric loss tangent (tan*δ_ε_)*; (**d**–**g**) the Cole-Cole semicircles of NC, Co/C, and DPC/CNTs-2, 6: (**h**,**i**) complex permittivity, (**j**) magnetic loss tangent (tan*δ_μ_*).

As we know, the complex permittivity (*ε_r_* = *ε*′ − j*ε*″) and permeability (*μ_r_* = *μ*′ − j*μ*″) are crucial for the MA materials. In general, the real part values of both are mainly associated with the electrical and magnetic energy storage capacity of the material, while the imaginary part value corresponds to the dissipation capacity of electrical and magnetic energy [53,54]. As displayed in Figure 5a,b, the real and imaginary parts of the permittivity for all samples exhibit a decreasing trend with increasing frequency, showing typical dispersive behaviour, which is often attributed to dipole orientation polarization [55,56]. Meanwhile, the DPC/CNTs show higher *ε*′ and *ε*″ values compared to the NC and Co/C, indicating the stronger storage and loss capacity for electromagnetic waves. The increase in the complex permittivity of the materials is attributable to the significant enhancement of interfacial polarization and conductive loss. The interfacial polarization is due to the microstructure of the 2D leafy carbon framework, and the Co atoms induced carbon nanotubes, leading to the accumulation of electrons at numerous carbon–air and carbon–carbon heterojunction interfaces. Conductivity loss is caused by the good conductivity of carbon frameworks and carbon nanotubes. As shown in Figure 5c, the loss tangent of the dielectric for DPC/CNTs is greater than that of NC and Co/C, further confirming the enhanced microwave attenuation ability of the 2D leafy porous carbon/carbon nanotubes heterostructure. The variation trend in the dielectric constant for DPC/CNTs with different ZIF-67 loading amounts (and the quantity of carbon nanotubes on the surface of the leafy carbon framework) also supports the above results (Figure 5a,b). It is worth noting that when the ZIF-67 loading amount reaches 6 mmol, the loss tangent of the dielectric shows a decreasing trend, which may be due to the more densely packed carbon nanotube, which does not promote the generation of interfacial polarization [57].

Figure 5d–g shows the Cole curves of the NC, Co/C, and DPC/CNTs. Following Debye’s theory, the relation between *ε*′ and *ε*″ is shown in Equation (3):

(3) $${\left[\varepsilon^{\prime }-\frac{1}{2}\left(\varepsilon_{s}+\varepsilon_{\infty }\right)\right]}^{2}+{\left(\varepsilon^{\prime \prime }\right)}^{2}=\frac{1}{4}{\left(\varepsilon_{s}+\varepsilon_{\infty }\right)}^{2}$$

When *ε′* and *ε″* meet Equation (3), the *ε′-ε″* plot will show a single semicircle, denoting the de-equilibrium domain procedure [58]. From Figure 5d–g, it can be seen that there are many Cole semicircles in most all of the samples except for Co/C, which is in good agreement with the resonance peaks on the complex dielectric constant curves. Compared to the NC and Co/C, the DPC/CNTs have more semicircles, along with the presence of a tail-up straight line at the end of the curve representing the conductive loss. These emphasize that DPC/CNTs have stronger interfacial polarization strength and higher conductive loss.

**Figure 6:**
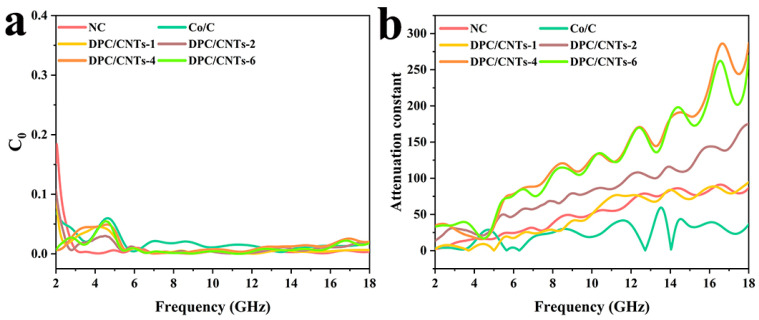
(**a**) the *C*_0_ values and (**b**) the attenuation constant α.

Permeability is another important parameter that affects the performance of MA, in the same way as the dielectric constant. As seen in Figure 5h–j, within the range of 6–18 GHz, the *μ′* and *μ″* of all samples vary between 1.0–1.2 and 0–0.4, respectively. The corresponding average tan*δ_μ_* is around 0.2, which is significantly smaller than the tan*δ_ε_*, indicating that the dielectric loss is the primary factor influencing the MA property. Magnetic loss is typically attributed to eddy current effects and the natural and exchange resonances. Multiple resonance peaks in the *μ″* curve suggest the presence of natural and exchange resonances, diminishing the electromagnetic energy. Concerning the eddy current effects, if the value of *C*_0_ (*C*_0_ = *μ″ (μ′)* − 2*f*^−1^) remains constant, magnetic loss will occur [59]. As shown in Figure 6a, the fluctuating nature of *C*_0_ for all the samples in the range of 6 to 18 GHz indicates the presence of eddy current losses [60].

The attenuation constant (*α*) represents the overall ability to dissipate incident electromagnetic waves and is calculated as the sum of the dielectric and magnetic losses. The formula is shown below [61]:

(4) $$\alpha =\frac{\sqrt{2}\pi f}{c}\sqrt{\left(\mu^{\prime \prime }\varepsilon^{\prime \prime }-\mu^{\prime }\varepsilon^{\prime }\right)+\sqrt{{\left(\mu^{\prime \prime }\varepsilon^{\prime \prime }-\mu^{\prime }\varepsilon^{\prime }\right)}^{2}+{\left(\mu^{\prime }\varepsilon^{\prime \prime }+\mu^{\prime \prime }\varepsilon^{\prime }\right)}^{2}}}$$

As demonstrated in Figure 6b, the alpha curves of all samples display a comparable pattern at the examined frequencies. Specifically, due to relatively weak dielectric and magnetic loss capabilities, NC and Co/C have the lowest *α* values among these six samples. For the DPC/CNTs composites, the trend of attenuation constant with the change in the ZIF-67 loading is consistent with the *ε″* values, further confirming the predominant role of the dielectric loss in the attenuation mechanism of DPC/CNTs. The continuously increasing attenuation constant also explains the excellent high-frequency MA performance of the DPC/CNTs composites.

**Figure 7:**
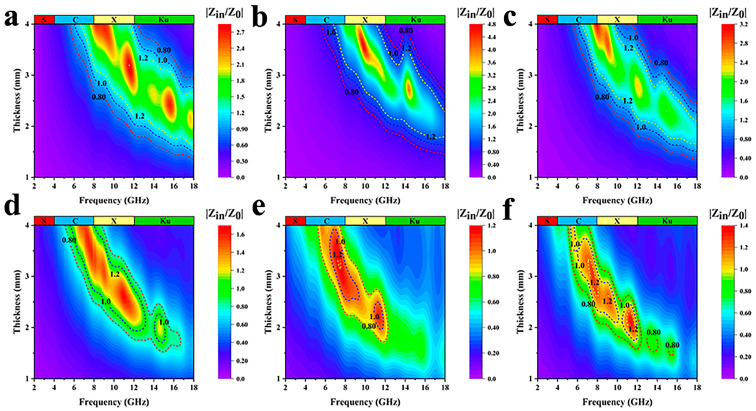
Two-dimensional contour maps of Z values for (**a**) NC, (**b**) ZIF-67, and (**c**–**f**) DPC-CNTs-1, 2, 4, 6.

In addition to the value of *α*, impedance matching (*Z* = *|Z_in_/Z*_0_*|*) is another important factor influencing the MA performance of materials. Poor impedance matching can lead to microwave penetration through the sample or reflection at the sample surface, thereby reducing the MA performance. When the *Z* value equals 1, electromagnetic waves can enter the absorber with zero reflection. Figure 7 presents a two-dimensional contour plot of the *Z* values for the samples. The figure shows the optimal impedance matching position with blue dashed lines. At this position, the *Z* value equals 1, indicating zero reflection of the incident microwave into the absorber. From the figure, we can easily see that the thermally collapsed morphologies of the NC, Co/C, and DPC/CNTs-1 have higher Z values, indicating poor impedance matching and high reflectivity at the interface between air and the materials, resulting in poor absorption performance. When the ZIF-L surface is fully covered by ZIF-67, the *Z* value significantly decreases (Figure 7d–f). However, it is important to note that although the *Z* values of DPC/CNTs-4 and DPC/CNTs-6 are nearer to 1 within the low-frequency range, the attenuation constants of the materials are smaller at this point; thus, good MA performance cannot be achieved. In contrast, the DPC/CNTs-2 exhibits the best MA performance in the high-frequency range by having both a Z-value close to 1 and a higher α value. Additionally, the design of efficient MA materials should follow the λ/4 rule. Based on the quarter-wavelength theory, the relationship between the peak frequency of the DPC/CNTs-2 reflection absorption and the quarter-wavelength is studied. The absorbent thickness (*t_m_)* and peak frequency (*f_m_*) satisfy the following Equation [62]:

(5) $$t_{m}=\frac{n\lambda }{4}=\frac{nc}{4f_{m}\sqrt{\left|u_{r}\varepsilon_{r}\right|}}\;(n=1,3,5\ldots )$$

It can be observed that the t_m_ and quarter-wavelength curves match well, and when the RL_min_ achieves −42.19 dB, |*Z_i_*_n_/*Z*_0_| is precisely equal to 1.0 (Figure S5). Therefore, outstanding MA performance is the outcome of the combined effects of the appropriate impedance matching and significant attenuation loss.

**Figure 8:**
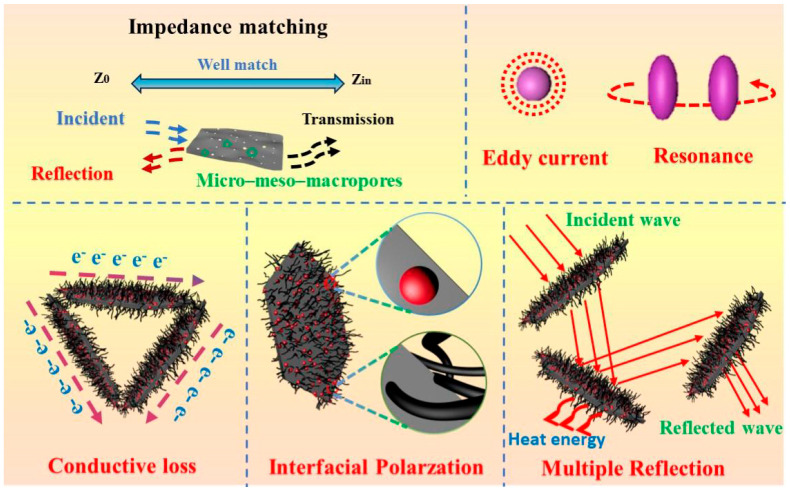
Schematic representation of the possible MA mechanism for DPC/CNTs composites.

Figure 8 provides an overview of the MA mechanism of the layered spiral DPC/CNTs composites. Firstly, electromagnetic waves are reflected, transmitted, or absorbed when they reach the surface of the microwave absorber. The abundant microporous structure and high specific surface area can effectively reduce the complex dielectric constant, enhancing the impedance matching of the MA materials, allowing more microwaves to pass into the material and broaden its absorption bandwidth [43]. Secondly, the Co atoms present in the material contribute to eddy current loss and resonance loss [63,64]. Thirdly, the uniformly sized carbon-based matrix layers overlap to form a conductive network, facilitating electron transfer and converting electromagnetic energy into heat, enhancing the material’s dielectric loss [65]. Fourth, the interface between the Co ions and self-generated carbon nanotubes with the carbon matrix promotes the generation of interface polarization [66]. Finally, multiple reflections occur between the layers and within the mesopores, thereby enhancing its attenuation ability [55].

**Figure 9:**
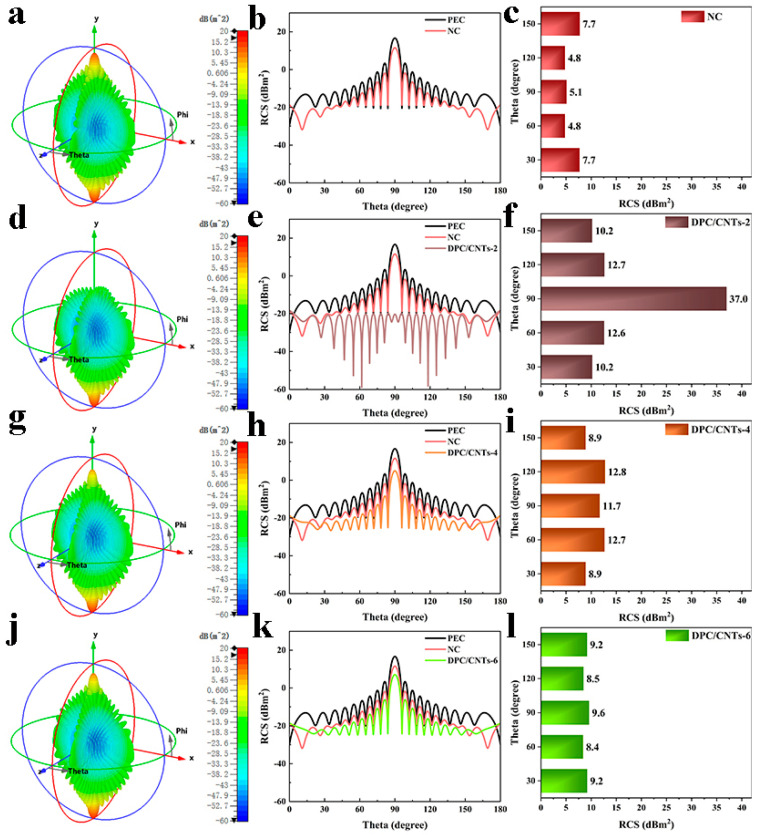
(**a**,**d**,**g**,**j**) CST simulation results based on the far-field response of NC, DPC/CNTs-2, DPC/CNTs-4, and DPC/CNTs-6. (**b**,**e**,**h**,**k**) RCS simulated curves of NC, DPC/CNTs-2, DPC/CNTs-4, and DPC/CNTs-6. (**c**,**f**,**i**,**l**) RCS reductions achieved by subtracting the NC, DPC/CNTs-2, DPC/CNTs-4, and DPC/CNTs-6.

Radar Cross Section (RCS) is a physical parameter that analyses the material’s ability to scatter electromagnetic waves, aiding in the design of appropriate absorption structures and stealth devices. The RCS results were obtained by using electromagnetic simulations to verify the absorption performance of the microwave absorber. The simulation model was set up as follows: a perfect electrical conductor (PEC) plate covered with a layer of absorbing material on top and parallel to the XOZ plane was placed on the bottom layer. Both were 200 mm in length and width and 2.2 mm and 1 mm in thickness, respectively. Generally, the RCS value could be influenced by factors like the incident angle of electromagnetic waves, polarization, the structure of the object, and the material type. Theoretical values can be calculated through the RCS to verify the performance of new materials [67]. We simulated the RCS distribution of the four samples using CST Microwave Studio. Figure 9a,d,g,j show the 3D RCS distribution of the models at varying angles of detection. It can be seen that the reflection loss values of all four samples are lower than a simple perfect electric conductor (PEC) layer (Figure S6), with the DPC/CNTs-2 having the lowest reflection loss. The 2D RCS curves on the x-o-y plane for different samples are presented in Figure 9b,e,h,k. From the graphs, it is evident that the DPC/CNTs exhibit a smaller RCS value compared to the NC, which experienced significant collapse in the thermal morphology. Additionally, at *θ* = 90°, the corresponding RCS reduction in the DPC/CNTs-2 can reach 37 dBm^2^, far higher than DPC/CNTs-4 at 11.7 and DPC/CNTs-6 at 9.6 (Figure 9c,f,i,l). This further indicates that DPC/CNTs-2 possesses outstanding electromagnetic energy absorption performance. The simulation results demonstrate that DPC/CNTs-2 can more effectively reduce radar reflection signals, suggesting its feasibility for commercial implementation in the field of MA.

## 4. Conclusions

Utilizing the MOF-on-MOF method, ZIF-67 was uniformly loaded onto ZIF-L, followed by high-temperature annealing in a nitrogen-protected atmosphere to obtain a two-dimensional leaf-like Co-doped porous carbon/carbon nanotube heterostructure. A series of DPC/CNTs composites were obtained by varying the amount of ZIF-67. The results indicate that maintaining the leaf-like structure and generating surface-disordered carbon nanotubes are crucial for adjusting the material’s dielectric performance. With an increase in the loading amount of ZIF-67, the dielectric loss performance of the material initially increases and then decreases. Among them, the DPC/CNTs-4 sample obtained the lowest RL value (−59.8 dB), and the DPC/CNTs-2 sample, under a thickness of 2.2 mm, exhibited an RL value of −42.2 dB with an EAB of 4.8 GHz. The study shows that DPC/CNTs composites possess lightweight and high-performance MA properties. This indicates wide prospects for 2D MOFs-derived carbon-based MA materials.

## Data Availability

Data are contained within the article and Supplementary Materials.

## Supplementary Materials

The following supporting information can be downloaded at: https://www.mdpi.com/article/10.3390/molecules29112426/s1.

## References

[1] He L., Xu J., Zhang N., Xue S., Wang X., Jin Q. (2023). Hollow multi-shelled structured BaTiO_3_/Fe_3_O_4_ composite: Confined space and interface effect with boosted microwave absorption. Ceram. Int..

[2] He L., He L., Xu H., Wang X., Jin Q. (2024). Space-Confined multiple interface in super-structured TiO_2_@PPy composite for enhanced electromagnetic wave absorption. Appl. Surf. Sci..

[3] Yin P., Zhang L., Wang J., Feng X., Zhao L., Rao H., Wang Y., Dai J. (2019). Preparation of SiO_2_-MnFe_2_O_4_ Composites via One-Pot Hydrothermal Synthesis Method and Microwave Absorption Investigation in S-Band. Molecules.

[4] Sun J., Li L., Yu R., Ma X., Jin S., Chen K., Chen S., Lv X., Shu Q. (2020). Synthesis and Microwave Absorption Properties of Sulfur-Free Expanded Graphite/Fe_3_O_4_ Composites. Molecules.

[5] Lu Y., Yang P., Li Y., Wen D., Luo J., Wang S., Wu F., Fang L., Pang Y. (2021). A Facile Synthesis of NiFe-Layered Double Hydroxide and Mixed Metal Oxide with Excellent Microwave Absorption Properties. Molecules.

[6] Yan J., Wang Y., Liu W., Liu P., Chen W. (2023). Two-Dimensional Metal Organic Framework derived Nitrogen-doped Graphene-like Carbon Nanomesh toward Efficient Electromagnetic Wave Absorption. J. Colloid Interface Sci..

[7] Zhang Y., Zhang H.-B., Wu X., Deng Z., Zhou E., Yu Z.-Z. (2019). Nanolayered Cobalt@Carbon Hybrids Derived from Metal–Organic Frameworks for Microwave Absorption. ACS Appl. Nano Mater..

[8] Qu Z., Wang Y., Yang P., Zheng W., Li N., Bai J., Zhang Y., Li K., Wang D., Liu Z. (2021). Enhanced Electromagnetic Wave Absorption Properties of Ultrathin MnO_2_ Nanosheet-Decorated Spherical Flower-Shaped Carbonyl Iron Powder. Molecules.

[9] Wang Y.-Y., Sun W.-J., Yan D.-X., Dai K., Li Z.-M. (2021). Ultralight carbon nanotube/graphene/polyimide foam with heterogeneous interfaces for efficient electromagnetic interference shielding and electromagnetic wave absorption. Carbon.

[10] Cruz-Martinez H., Garcia-Hilerio B., Montejo-Alvaro F., Gazga-Villalobos A., Rojas-Chavez H., Sanchez-Rodriguez E.P. (2024). Density Functional Theory-Based Approaches to Improving Hydrogen Storage in Graphene-Based Materials. Molecules.

[11] Gu W., Shi J., Zhang J., Jia Q., Liu C., Ge H., Sun Q., Zhu L. (2023). Fabrication and Investigation of the Microwave Absorption of Nonwovens Modified by Carbon Nanotubes and Graphene Flakes. Molecules.

[12] Abutaleb A., Imran M., Zouli N., Khan A.H., Hussain S., Ali M.A., Bakather O., Gondal M.A., Khan N.A., Panchal H. (2023). Fe_3_O_4_-multiwalled carbon nanotubes-bentonite as adsorbent for removal of methylene blue from aqueous solutions. Chemosphere.

[13] Wu D., Jiang J., Deng S., He Q., Wang Y. (2023). Rational construction of mushroom-like Ni@N-doped carbon tubes composites with enhanced electromagnetic wave absorption. J. Alloys Compd..

[14] Peng S., Wang S., Hao G., Zhu C., Zhang Y., Lv X., Hu Y., Jiang W. (2019). Preparation of magnetic flower-like carbon-matrix composites with efficient electromagnetic wave absorption properties by carbonization of MIL-101(Fe). J. Magn. Magn. Mater..

[15] Li L., Li G., Ouyang W., Zhang Y., Zeng F., Liu C., Lin Z. (2021). Bimetallic MOFs derived FeM(II)-alloy@C composites with high-performance electromagnetic wave absorption. Chem. Eng. J..

[16] Ma J., Liu W., Liang X., Quan B., Cheng Y., Ji G., Meng W. (2017). Nanoporous TiO_2_/C composites synthesized from directly pyrolysis of a Ti-based MOFs MIL-125(Ti) for efficient microwave absorption. J. Alloys Compd..

[17] Wang K., Chen Y., Tian R., Li H., Zhou Y., Duan H., Liu H. (2018). Porous Co-C Core-Shell Nanocomposites Derived from Co-MOF-74 with Enhanced Electromagnetic Wave Absorption Performance. ACS Appl. Mater. Interfaces.

[18] Guo Y., Wang D., Wang J., Tian Y., Liu H., Liu C., Shen C. (2022). Hierarchical HCF@NC/Co Derived from Hollow Loofah Fiber Anchored with Metal-Organic Frameworks for Highly Efficient Microwave Absorption. ACS Appl. Mater. Interfaces.

[19] Xu X., Ran F., Fan Z., Cheng Z., Lv T., Shao L., Xie Z., Liu Y. (2021). Acidified bimetallic MOFs constructed Co/N co-doped low dimensional hybrid carbon networks for high-efficiency microwave absorption. Carbon.

[20] Bi Y., Ma M., Liu Y., Tong Z., Wang R., Chung K.L., Ma A., Wu G., Ma Y., He C. (2021). Microwave absorption enhancement of 2-dimensional CoZn/C@MoS_2_@PPy composites derived from metal-organic framework. J. Colloid Interface Sci..

[21] Zou L., Zhong G., Nie Y., Tan Z., Liao W., Fu X., Pan Z. (2021). Porous Carbon Nanosheets Derived from ZIF-8 Treated with KCl as Highly Efficient Electrocatalysts for the Oxygen Reduction Reaction. Energy Technol..

[22] Yang K., Long L., Feng Y., Wei Y., Wu T., Gao Z., Zhang J. (2022). Tunable regulation of metal-semiconductor heterostructures toward Ag/ZnO hybrids for electromagnetic wave absorption. J. Alloys Compd..

[23] Lee S., Oh S., Oh M. (2020). Atypical Hybrid Metal-Organic Frameworks (MOFs): A Combinative Process for MOF-on-MOF Growth, Etching, and Structure Transformation. Angew. Chem..

[24] Liu C., Lin L., Sun Q., Wang J., Huang R., Chen W., Li S., Wan J., Zou J., Yu C. (2020). Site-specific growth of MOF-on-MOF heterostructures with controllable nano-architectures: Beyond the combination of MOF analogues. Chem. Sci..

[25] Pan J., Xia W., Sun X., Wang T., Li J., Sheng L., He J. (2020). Improvement of interfacial polarization and impedance matching for two-dimensional leaf-like bimetallic (Co, Zn) doped porous carbon nanocomposites with broadband microwave absorption. Appl. Surf. Sci..

[26] Liang X., Quan B., Ji G., Liu W., Cheng Y., Zhang B., Du Y. (2016). Novel nanoporous carbon derived from metal–organic frameworks with tunable electromagnetic wave absorption capabilities. Inorg. Chem. Front..

[27] Wen H., Qu W., Lin M., Zhou L., Guo X., Ma P., Wu T., Zhao H., Zhong T., He C. (2023). Nitrogen-coordinated cobalt embedded in hollow carbon polyhedron for catalytic ozonation of odor CH_3_SH at ambient temperature. Chem. Eng. J..

[28] Yin Y., Liu X., Wei X., Yu R., Shui J. (2016). Porous CNTs/Co Composite Derived from Zeolitic Imidazolate Framework: A Lightweight, Ultrathin, and Highly Efficient Electromagnetic Wave Absorber. ACS Appl. Mater. Interfaces.

[29] Meng J., Niu C., Xu L., Li J., Liu X., Wang X., Wu Y., Xu X., Chen W., Li Q. (2017). General Oriented Formation of Carbon Nanotubes from Metal-Organic Frameworks. J. Am. Chem. Soc..

[30] Xu X., Ran F., Fan Z., Lai H., Cheng Z., Lv T., Shao L., Liu Y. (2019). Cactus-Inspired Bimetallic Metal-Organic Framework-Derived 1D-2D Hierarchical Co/N-Decorated Carbon Architecture toward Enhanced Electromagnetic Wave Absorbing Performance. ACS Appl. Mater. Interfaces.

[31] Zhang Y., Wu J., Zhang S., Shang N., Zhao X., Alshehri S.M., Ahamad T., Yamauchi Y., Xu X., Bando Y. (2022). MOF-on-MOF nanoarchitectures for selectively functionalized nitrogen-doped carbon-graphitic carbon/carbon nanotubes heterostructure with high capacitive deionization performance. Nano Energy.

[32] Feng Y., Wang H., Yao J. (2021). Synthesis of 2D nanoporous zeolitic imidazolate framework nanosheets for diverse applications. Coord. Chem. Rev..

[33] Zhang X., Qiao J., Jiang Y., Wang F., Tian X., Wang Z., Wu L., Liu W., Liu J. (2021). Carbon-Based MOF Derivatives: Emerging Efficient Electromagnetic Wave Absorption Agents. Nano-Micro Lett..

[34] Parsapour F., Moradi M., Bahadoran A. (2023). Metal-organic frameworks-derived layered double hydroxides: From controllable synthesis to various electrochemical energy storage/conversion applications. Adv. Colloid Interface Sci..

[35] Song A., Yang W., Yang W., Sun G., Yin X., Gao L., Wang Y., Qin X., Shao G. (2017). Facile Synthesis of Cobalt Nanoparticles Entirely Encapsulated in Slim Nitrogen-Doped Carbon Nanotubes as Oxygen Reduction Catalyst. ACS Sustain. Chem. Eng..

[36] Yang L., Wang Y., Lu Z., Cheng R., Wang N., Li Y. (2023). Construction of multi-dimensional NiCo/C/CNT/rGO aerogel by MOF derivative for efficient microwave absorption. Carbon.

[37] Wang Y.-Y., Zhu J.-L., Li N., Shi J.-F., Tang J.-H., Yan D.-X., Li Z.-M. (2022). Carbon aerogel microspheres with in-situ mineralized TiO2 for efficient microwave absorption. Nano Res..

[38] Li J., Wu Q., Wang X., Wang B., Liu T. (2022). Metal-organic framework-derived Co/CoO nanoparticles with tunable particle size for strong low-frequency microwave absorption in the S and C bands. J. Colloid Interface Sci..

[39] Wang X., Bao X., Zhou X., Shi G. (2018). Excellent microwave absorption of lamellar LaOCl/C nanocomposites with LaOCl nanoparticles embedded in carbon matrix. J. Alloys Compd..

[40] Khan A., Cong J., Kumar R.R., Ahmed S., Yang D., Yu X. (2022). Chemical Vapor Deposition of Graphene on Self-Limited SiC Interfacial Layers Formed on Silicon Substrates for Heterojunction Devices. ACS Appl. Nano Mater..

[41] Khan A., Habib M.R., Jingkun C., Xu M., Yang D., Yu X. (2021). New Insight into the Metal-Catalyst-Free Direct Chemical Vapor Deposition Growth of Graphene on Silicon Substrates. J. Phys. Chem. C.

[42] Wang W., Ye W., Hou X., Ran K., Huang Y., Zhang Z., Fang Y., Wang S., Zhao R., Xue W. (2023). Salt-assisted pyrolysis of carbon nanosheet and carbon nanoparticle hybrids for efficient microwave absorption. J. Mater. Chem. C.

[43] Zhou Y., Wang H., Wang D., Yang X., Xing H., Feng J., Zong Y., Zhu X., Li X., Zheng X. (2023). Insight to the enhanced microwave absorption of porous N-doped carbon driven by ZIF-8: Competition between graphitization and porosity. Int. J. Miner. Metall. Mater..

[44] Imran M., Zouli N., Ahamad T., Alshehri S.M., Chandan M.R., Hussain S., Aziz A., Dar M.A., Khan A. (2021). Carbon-coated Fe_3_O_4_ core-shell super-paramagnetic nanoparticle-based ferrofluid for heat transfer applications. Nanoscale Adv..

[45] Liang X., Wang G., Gu W., Ji G. (2021). Prussian blue analogue derived carbon-based composites toward lightweight microwave absorption. Carbon.

[46] Quan B., Xu G., Yi H., Yang Z., Xiang J., Chen Y., Ji G. (2018). Enhanced electromagnetic wave response of nickel nanoparticles encapsulated in nanoporous carbon. J. Alloys Compd..

[47] Wang Y., Wang H., Ye J., Shi L., Feng X. (2020). Magnetic CoFe alloy@C nanocomposites derived from ZnCo-MOF for electromagnetic wave absorption. Chem. Eng. J..

[48] Liao Q., He M., Zhou Y., Nie S., Wang Y., Wang B., Yang X., Bu X., Wang R. (2018). Rational Construction of Ti_3_C_2_T _x_/Co-MOF-Derived Laminated Co/TiO_2_-C Hybrids for Enhanced Electromagnetic Wave Absorption. Langmuir.

[49] Lu Y., Wang Y., Li H., Lin Y., Jiang Z., Xie Z., Kuang Q., Zheng L. (2015). MOF-Derived Porous Co/C Nanocomposites with Excellent Electromagnetic Wave Absorption Properties. ACS Appl. Mater. Interfaces.

[50] Wang H., Xiang L., Wei W., An J., He J., Gong C., Hou Y. (2017). Efficient and Lightweight Electromagnetic Wave Absorber Derived from Metal Organic Framework-Encapsulated Cobalt Nanoparticles. ACS Appl. Mater. Interfaces.

[51] Zhao Z., Xu S., Du Z., Jiang C., Huang X. (2019). Metal–Organic Framework-Based PB@MoS_2_ Core–Shell Microcubes with High Efficiency and Broad Bandwidth for Microwave Absorption Performance. ACS Sustain. Chem. Eng..

[52] Zhang X., Ji G., Liu W., Zhang X., Gao Q., Li Y., Du Y. (2016). A novel Co/TiO_2_ nanocomposite derived from a metal–organic framework: Synthesis and efficient microwave absorption. J. Mater. Chem. C.

[53] Huang J., Gu H., Li N., Yang H., Chen G., Zhang L., Dong C., Guan H. (2022). Polypyrrole/Schiff Base Composite as Electromagnetic Absorbing Material with High and Tunable Absorption Performance. Molecules.

[54] Sun Q., Yang X., Shu T., Yang X., Qiao M., Wang D., Liu Z., Li X., Rao J., Zhang Y. (2022). In Situ Synthesis of C-N@NiFe_2_O_4_@MXene/Ni Nanocomposites for Efficient Electromagnetic Wave Absorption at an Ultralow Thickness Level. Molecules.

[55] Xiang Z., Wang Y., Yin X., He Q. (2023). Microwave absorption performance of porous heterogeneous SiC/SiO_2_ microspheres. Chem. Eng. J..

[56] Huang Y., Xue W., Hou X., Zhao R. (2021). Metal Oxide/Nitrogen-Doped Carbon Nanosheet Heteronanostructures as Highly Efficient Electromagnetic Wave Absorbing Materials. Molecules.

[57] Xu X., Ran F., Lai H., Cheng Z., Lv T., Shao L., Liu Y. (2019). In Situ Confined Bimetallic Metal-Organic Framework Derived Nanostructure within 3D Interconnected Bamboo-like Carbon Nanotube Networks for Boosting Electromagnetic Wave Absorbing Performances. ACS Appl. Mater. Interfaces.

[58] Lv Y., Xiu T., Zhang Y., Zhang B. (2024). Controlled fabrication and microwave absorption performance of cucurbit-like carbon nanofibers. Diam. Relat. Mater..

[59] Wang X., Geng Q., Shi G., Xu G., Yu J., Guan Y., Zhang Y., Li D. (2019). One-pot solvothermal synthesis of Fe/Fe_3_O_4_ composites with broadband microwave absorption. J. Alloys Compd..

[60] Wang L., Zhu S., Zhu J. (2022). Constructing ordered macropores in hollow Co/C polyhedral nanocages shell toward superior microwave absorbing performance. J. Colloid Interface Sci..

[61] Liu Y., Zhang B., Yuan Y., Liu W., Wu H., Qin S., Fang Q., Yu G., Wang B. (2024). Kapok derived microtubular FeCo/CMT with high specific surface area for broadband microwave absorption. Ceram. Int..

[62] Liu Z., Wang K., Zhang H., Huang S., Xie N., Wang Q., Zhu H., Liu Y., Zhao X. (2024). Solvothermally Synthesized Co(CoO)/Ti_3_C_2_T_x_/TiO_2_ Nanocomposites for Enhanced Microwave Absorption. ACS Appl. Nano Mater..

[63] Li Z., Liang J., Wei Z., Cao X., Shan J., Li C., Chen X., Zhou D., Xing R., Luo C. (2024). Lightweight foam-like nitrogen-doped carbon nanotube complex achieving highly efficient electromagnetic wave absorption. J. Mater. Sci. Technol..

[64] Ge J., Cui Y., Qian J., Liu L., Meng F., Wang F. (2022). Morphology-controlled CoNi/C hybrids with bifunctions of efficient anti-corrosion and microwave absorption. J. Mater. Sci. Technol..

[65] Gu H., Huang J., Li N., Yang H., Chen G., Dong C., Gong C., Guan H. (2023). Reactive MnO_2_ template-assisted synthesis of double-shelled PPy hollow nanotubes to boost microwave absorption. J. Mater. Sci. Technol..

[66] Jiao Z., Hu J., Ma M., Liu Y., Zhao J., Wang X., Luan S., Zhang L. (2023). One-dimensional core-shell CoC@CoFe/C@PPy composites for high-efficiency microwave absorption. J. Colloid Interface Sci..

[67] Chen J., Zheng J., Wang F., Huang Q., Ji G. (2021). Carbon fibers embedded with FeIII-MOF-5-derived composites for enhanced microwave absorption. Carbon.

