# Supplementary material for: Co-Doped Porous Carbon/Carbon Nanotube Heterostructures Derived from ZIF-L@ZIF-67 for Efficient Microwave Absorption
Source: Molecules. 2024 May 21;29(11):2426. doi: 10.3390/molecules29112426 (PMC11173442; doi:10.3390/molecules29112426)
Supplement: Supplementary file 1 [file molecules-29-02426-s001.zip › molecules-2985960-supplementary.pdf]

Supplementary Materials

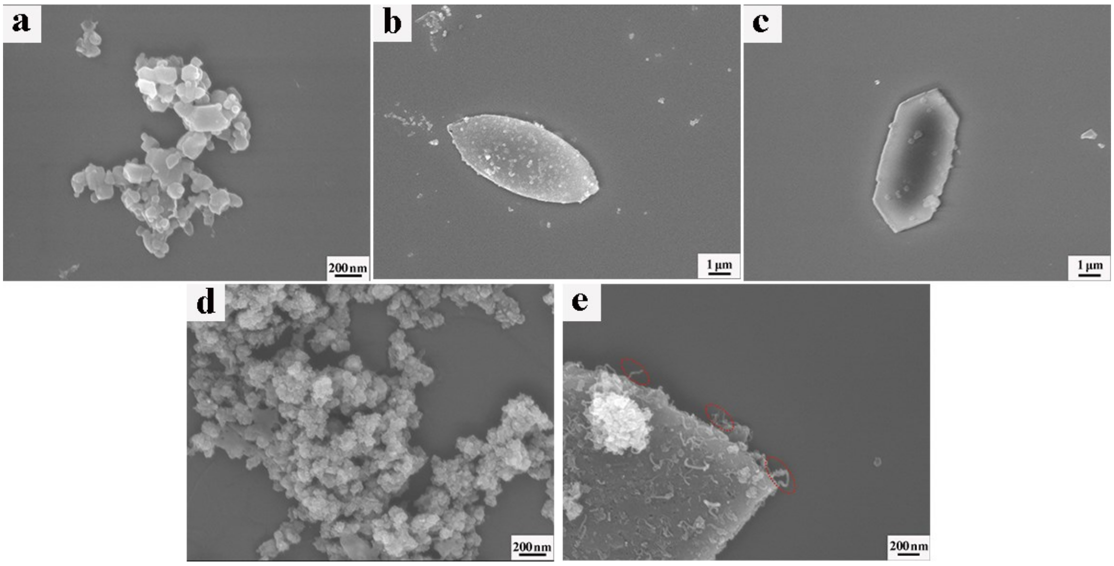

Figure S1. SEM of (**a**) ZIF-67 (**b**) ZIF-L@ZIF-67-1 (**c**) ZIF-L@ZIF-67-4 (**d**) Co/C (**e**) DPC/CNTs-4.

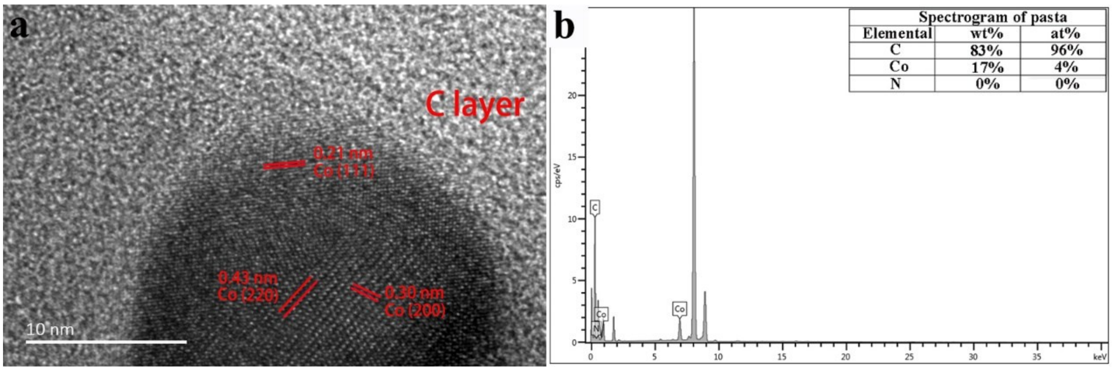

Figure S2. (**a**) HRTEM images of DPC/CNTs-2, (**b**) Elemental mapping of DPC/CNTs-2.

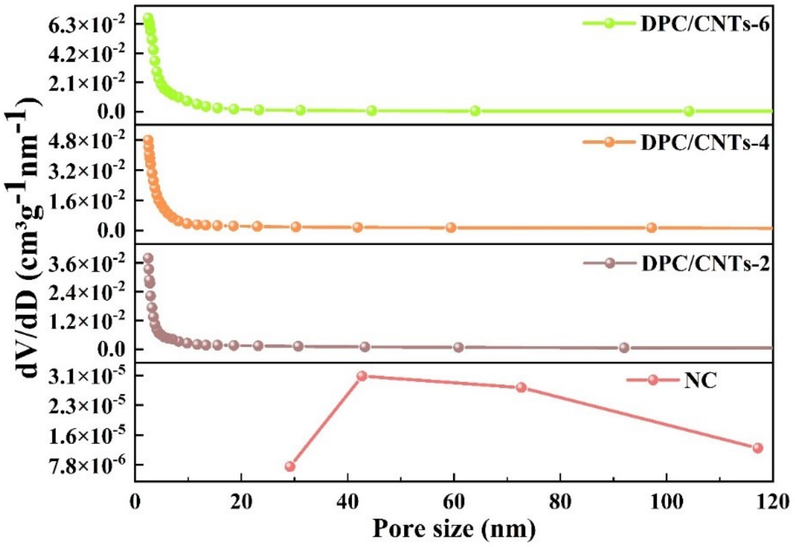

Figure S3. pore size distribution profiles of NC, DPC/CNTs-2, DPC/CNTs-4, DPC/CNTs-6.

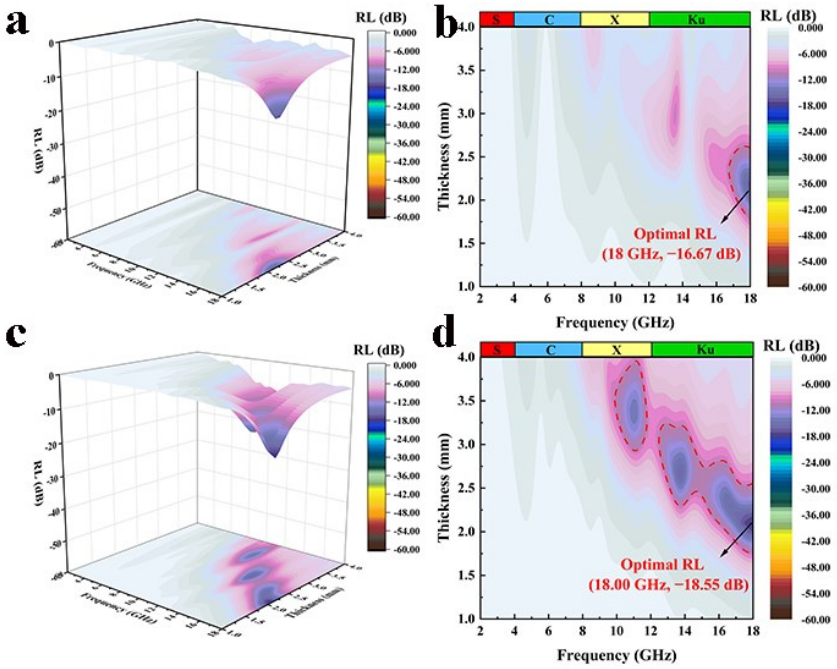

Figure S4. The RL and EAB values for (**a**,**b**) Co/C, (**c**,**d**) DPC/CNTs-1.

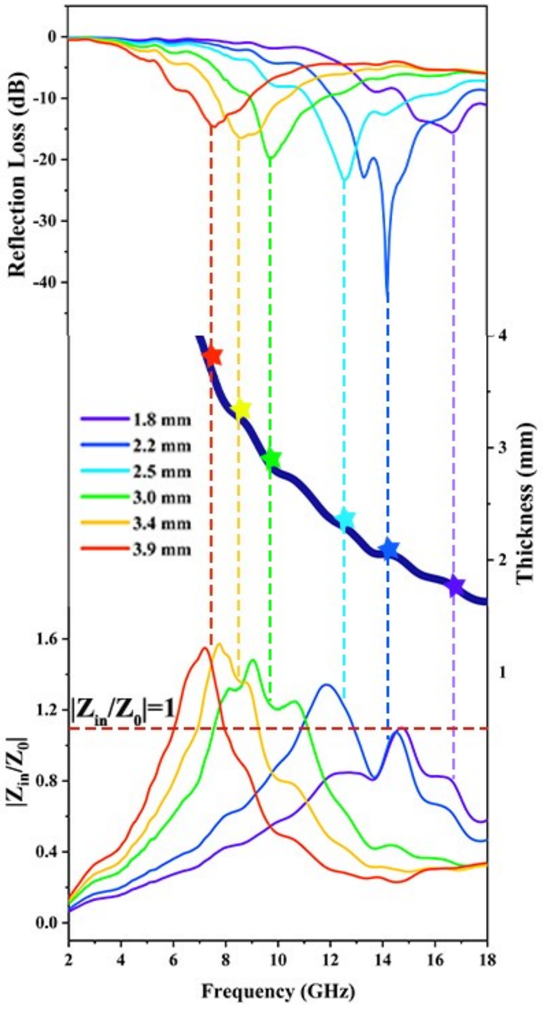

Figure S5. RL, t_m and $|Z_{in}/Z_0|$ vs f curves of DPC/CNTs-2.

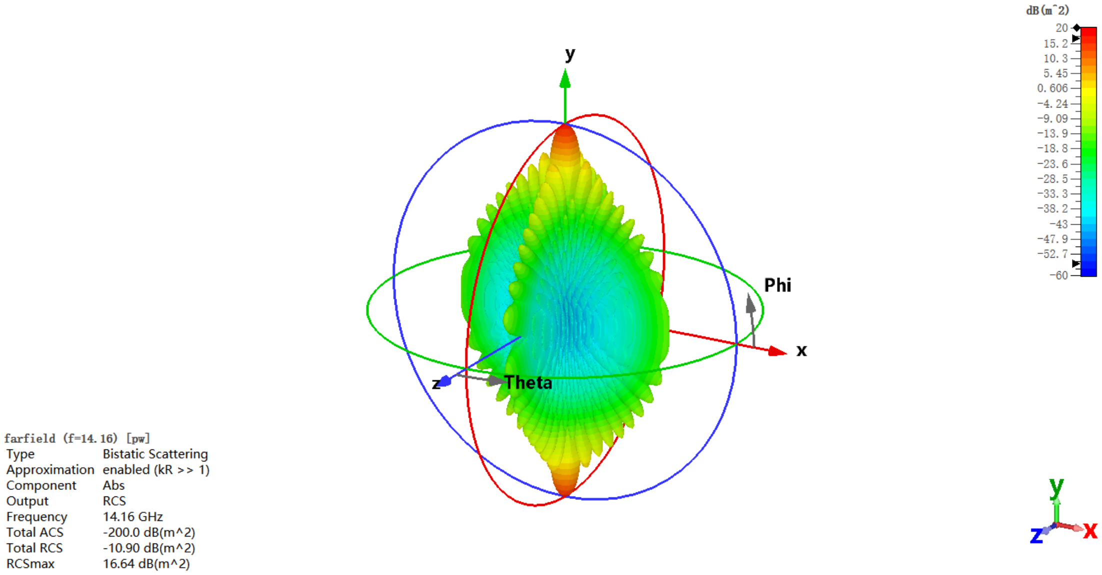

Figure S6. Simulation results based on far−field response of PEC.
